# Alexandra Hospital for Children with Hip Disease

**Published:** 1902-05-31

**Authors:** 


### ALEXANDRA HOSPITAL FOR CHILDREN WITH HIP DISEASE.

### ANNUAL MEETING.

"The proceedings will be purely formal" was the warning delivered by the secretary to the sole representative of the press who attended the meeting on May 22nd. This prediction was more than fulfilled; not only were the proceedings of a purely formal character, but they were transacted with a celerity that could only be described as breathless.

The committee issue an earnest appeal for help from all friends of the hospital in order to make good the serious decrease in donations from September 30th, 1901, to February 1902. The deficiency occasioned by this falling-off amounts to hundreds of pounds.

The meeting had been originally fixed for May 15th, but was postponed on account of an entertainment which was held at the hospital on that day. It was perhaps the alteration of date, combined with the heavy rain, which caused the small attendance.

